# A Higher Dose of *Staphylococcus aureus* Enterotoxin B Led to More Th1 and Lower Th2/Th1 Ratio in Th Cells

**DOI:** 10.3390/toxins15060363

**Published:** 2023-05-28

**Authors:** Jin Yuan, Xiaoqian Xu, Zhongliang Wang, Ping Tong, Xuanyi Meng, Yong Wu, Xin Li, Jinyan Gao, Hongbing Chen

**Affiliations:** 1 State Key Laboratory of Food Science and Technology, Nanchang University, Nanchang 330047, China; yuan2jin@email.ncu.edu.cn (J.Y.);; 2 China Sino-German Joint Research Institute, Nanchang University, Nanchang 330047, China; 3 College of Food Science & Technology, Nanchang University, Nanchang 330031, China

**Keywords:** *Staphylococcus aureus* enterotoxin B, T helper cell, naïve Th cell, cytokine, dendritic cell

## Abstract

Exposure to *Staphylococcus aureus* enterotoxin B (SEB) is one of the causes of food poisoning and is associated with several immune diseases due to its superantigen capability. This study aimed to characterize the differentiations of naïve Th cells stimulated with different doses of SEB. The expression of T-bet, GATA-3, and Foxp3 or secretion of IFN-γ, IL-4, IL-5, IL-13, and IL-10 were evaluated in wild-type (WT) or DO11.10 CD4 T cells co-cultured with bone marrow dendritic cells (BMDCs). We found that the balance of Th1/Th2 could be dominated by the doses of SEB stimulation. A higher SEB dose could induce more Th1 and a lower Th2/Th1 ratio in Th cells co-cultured with BMDCs. This different tendency of Th cell differentiation induced by the SEB complements the existing knowledge about SEB acting as a superantigen to activate Th cells. Additionally, it is also helpful in managing the colonization of *S. aureus* and food contamination of SEB.

## 1. Introduction

*Staphylococcus aureus* (*S. aureus*) is one of the most prevalent bacterial pathogens in foodborne diseases [1]. It was estimated that 30% to 40% of people are asymptomatically colonized by *S. aureus* on skin, nasal, gut, or other mucosal surfaces. Up to 70% of the population could be temporarily colonized [2]. The role of *S. aureus* in atopic dermatitis (AD) has been well expound, and nearly 100% of patients with AD are colonized with *S. aureus* on the skin [3]. Additionally, about 50–60% of individuals are intermittently or permanently colonized [4]. *S. aureus* is able to be a potent pathogen while simultaneously emerging as part of the normal flora, primarily in the secretion of pyrogenic toxin superantigens. *Staphylococcal* food poisoning, which leads to nausea, vomiting, abdominal cramping, and diarrhea, could be induced by consuming foods containing *Staphylococcus aureus* enterotoxins (SEs) [5]. In the food poisoning incidents caused by SEs, the content of SEB is 0.5–10 μg in 100 g of food. Poisoning symptoms could be induced by ingestion of foods containing 10–20 μg of SEB, some even less than 1 μg [6].

*Staphylococcus aureus* enterotoxin B (SEB) is one of the SEs and is also well known as a superantigen. SEB could bind directly to MHC-II molecules and TCR on dendritic cells (DCs) and T cells without antigens, thus bypassing the limitations of conventional antigen-activated T cells [7]. SEB may be ingested into the internal environment through contaminated food digestive tract, nasal, or conjunctival routes [8]. The abilities of SEB that traverse the intact intestinal or nasal epithelium and reduce the expression of the mucosal tight junction were demonstrated by cellular and in vivo models [9,10,11]. Lower SEB levels were detected in peripheral blood after oral or respiratory exposure due to the rapid binding of SEB to receptors [12]. Moreover, both types of exposure could elicit an acute systemic inflammatory response [13,14]. Exposure to SEB could lead to rapid activation of T cells and abnormal immune responses. This study examined the crystal structure of the superantigen. It determined that the superantigen contains a conserved overall system consisting of two major protein domains: an amino-terminated oligosaccharide/oligonucleotide binding fold composed of a barrel, and a carboxy-terminated grip domain consisting of antiparallel chains, a structure domain by a central diagonal screw connection [15].

Although it is an opportunistic pathogen, the prevalence of *S. aureus* and exposure to SEB are associated with several autoimmune-related diseases, such as AD, psoriasis, asthma, chronic rhinosinusitis, and food allergies [16,17]. In these diseases, the plentiful activation of T helper (Th) cells and secretion of cytokines induced by SEB are part of the critical pathogenicity [18,19,20]. According to previous research, all directions of Th differentiations could be caused by SEB [21,22,23]. In mouse models, administration of a higher concentration of the SEB may lead to suppression of Th immunity [22,24]. Additionally, the activation of Th2 cells in the high-dose SEB group was weaker than in the low-dose group [22,25]. We speculate that the dose of SEB might dominate the balance of Th differentiation. This work aimed to determine whether the dose of SEB could change the Th immunity balance, and thus if higher SEB leads to a lower Th2 activation.

Transcription factors, including T-bet, GATA-3, and Foxp3, regulate cell differentiation and control cell proliferation. T-bet could directly bind to the promoter of the *IFN-γ* gene and activate its transcription and act as an essential role in the differentiation of CD4 precursor cells into Th1 cells [26]. Similarly, GATA3 is essential for Th2 cell differentiation and secretion of Th2 factors such as IL-4, IL-5, and IL-13. Foxp3 regulatory T cells (Treg) are characterized by expressing main marks, including Foxp3, CD25, and TGF-β. Furthermore, the expression of T-bet, GATA-3, or RORγt in Tregs was described as Th-like Tregs that conditionally lost immune suppressive function and promoted the development of autoimmune diseases [27].

Previous studies have only fragmentary hints of SEB activation of Th1, Th2, or Treg and failed to explain why in some cases, the activation of Th2 was lower under stimulation of a high dose of SEB. A complete understanding of the dose effect of SEB on Th activation is challenging. To characterize the activation and balance of Th cells induced by different doses of SEB, 1 ng, 10 ng, and 100 ng of SEB were incorporated into the co-culture of bone marrow DCs (BMDCs) and naïve Th cells with a 1 mL total volume in this study. The expression of T-bet, GATA-3, CD25, and Foxp3 in Th cells was evaluated to characterize the differentiations of Th1, Th2, Treg, Th1-like Treg, and Th2-like Treg cells. We hope our results will benefit a more comprehensive understanding of the promotion of Th cell differentiation by SEB as a superantigen and its possible role in various immune diseases.

## 2. Results

### 2.1. Stimulating 1 ng/mL SEB Could Induce the Differentiation of Th2 and Treg Cells in Co-Culture with BMDCs

**Figure 1:**
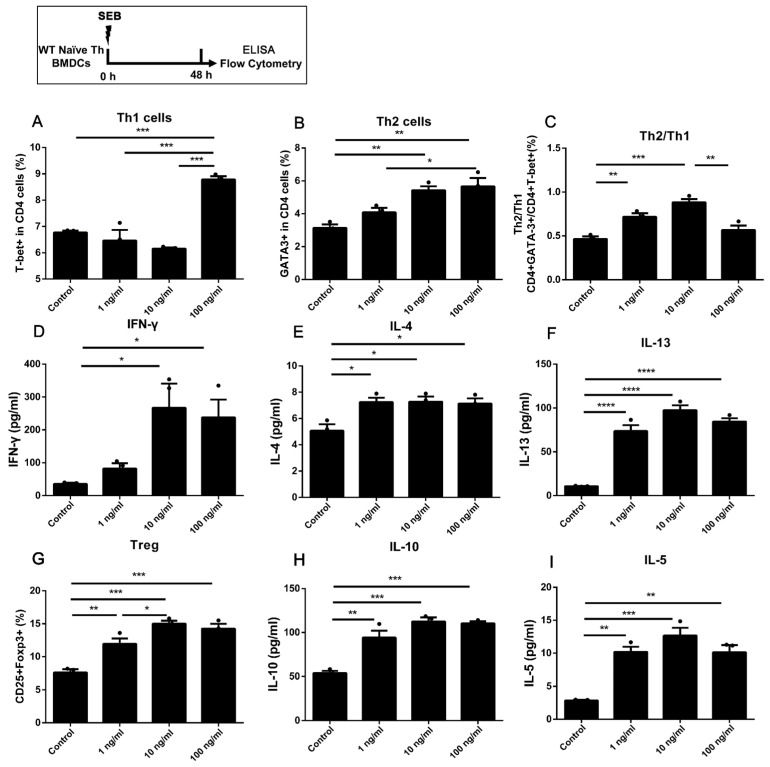
The differentiation in WT naïve Th cells co-cultured with BMDCs under stimulation of SEB. (**A**,**B**) The percentages of T-bet+ and GATA-3+ in CD4 T cells. (**C**), The ratio of GATA-3/T-bet in CD4 T cells. The levels of IFN-γ (**D**), IL-4 (**E**), and IL-13 (**F**) are shown. (**G**) The percentage of Foxp3+ CD25+ in CD4 T cells. The levels of IL-10 (**H**) and IL-5 (**I**) are also shown. Cells were stimulated with diluent (Control), 1 ng/mL SEB, 10 ng/mL SEB, and 100 ng/mL SEB (1 ng, 10 ng, or 100 ng SEB in each 1 mL medium) for 48 h. Data are represented with 3 technical replicates per group and presented as mean ± SEM. * *p* < 0.05, ** *p* < 0.01, *** *p* < 0.001, **** *p* < 0.0001.

Different doses of SEB (1, 10, and 100 ng/mL) were involved in a co-culture model of DCs and WT naïve T cells. The gating strategy of Th cells is shown in Figure S1A. A higher expression of T-bet in CD4 T cells could only be found in cells stimulated with a higher dose of SEB (100 ng/mL) compared to the Control group, with the up-regulated secretion of IFN-γ in the Middle (10 ng/mL) and High (100 ng/mL) groups (Figure 1A,D). The stimulation of middle and high doses of SEB (10 and 100 ng/mL) could also lead to higher expression of GATA-3 in CD4 T cells compared to the Control group (Figure 1B). The ratio of Th2/Th1 (GATA-3+/T-bet+ in CD4 cells) was higher in the 1 and 10 ng/mL groups than that in the Control (Figure 1C). The secretion of Th2 cytokines, including IL-4, IL-5, and IL-13, was significantly up-regulated in all SEB groups compared to the Control (Figure 1E,F,I). The proportion of Tregs (CD25+Foxp3+CD4 T cells) and secretion of IL-10 could be significantly increased under the stimulation of different doses of SEB compared to the Control (Figure 1G).

### 2.2. The Naïve Th Cells Alone Stimulated with a Higher Dose of SEB Tended to Have More Th1 and Less Th2 Differentiation

**Figure 2:**
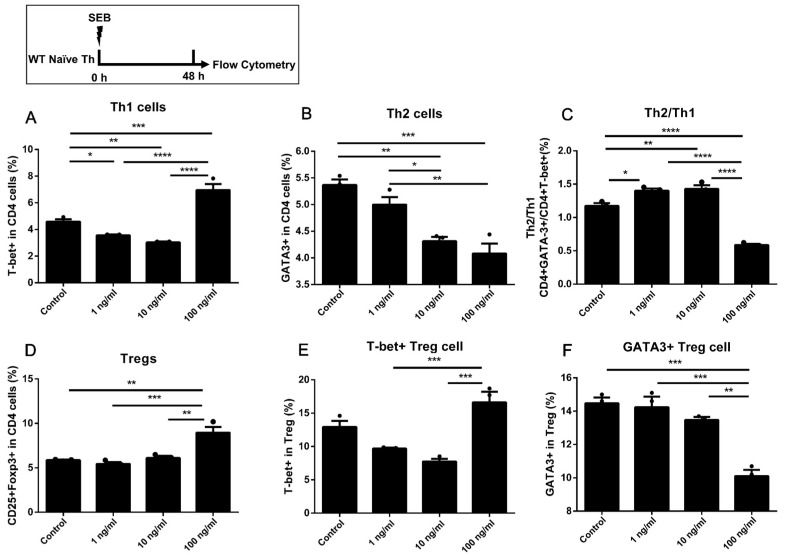
The differentiation in WT naïve Th cells stimulated with SEB. (**A**,**B**) The percentages of T-bet+ and GATA-3+ in CD4 T cell. (**C**) The ratio of GATA-3/T-bet in CD4 T cells. (**D**) The percentage of Foxp3+ CD25+ in CD4 T cells. (**E**,**F**) The percentages of T-bet+ and GATA-3+ in CD25+Foxp3+ CD4 T cells. Cells were stimulated with diluent (Control), 1 ng/mL SEB, 10 ng/mL SEB, and 100 ng/mL SEB (1 ng, 10 ng, or 100 ng SEB in each 1 mL medium) for 48 h. Data are represented with 3 technical replicates per group and presented as mean ± SEM. * *p* < 0.05, ** *p* < 0.01, *** *p* < 0.001, **** *p* < 0.0001.

The naïve Th cells were stimulated with SEB alone in Figure 2. A higher expression of T-bet in CD4 T cells could only be found in Th cells stimulated with 100 ng/mL SEB compared to the Control (Figure 2A). The expression of GATA-3 in CD4 T cells was decreased under the stimulation of 10 and 100 ng/mL SEB compared to the Control and 1 ng/mL group (Figure 2B). Moreover, the ratio of Th2/Th1 was significantly decreased in the 100 ng/mL group compared to the Control (Figure 2C). Only 100 ng/mL SEB led to a higher proportion of CD25+Foxp3+CD4 T cells than the Control (Figure 2D). The 100 ng/mL SEB stimulation increased the proportion of Th1-like Tregs compared to the Low and Middle groups (Figure 2E). The proportion of Th2-like Tregs in the 100 ng/mL group was lower than in other groups (Figure 2F).

### 2.3. Dose-Dependent Up-Regulation of Th1 and Treg in DO11.10 Naïve T Cells Could Be Induced by the Stimulation of SEB in Co-Culture with BMDCs

**Figure 3:**
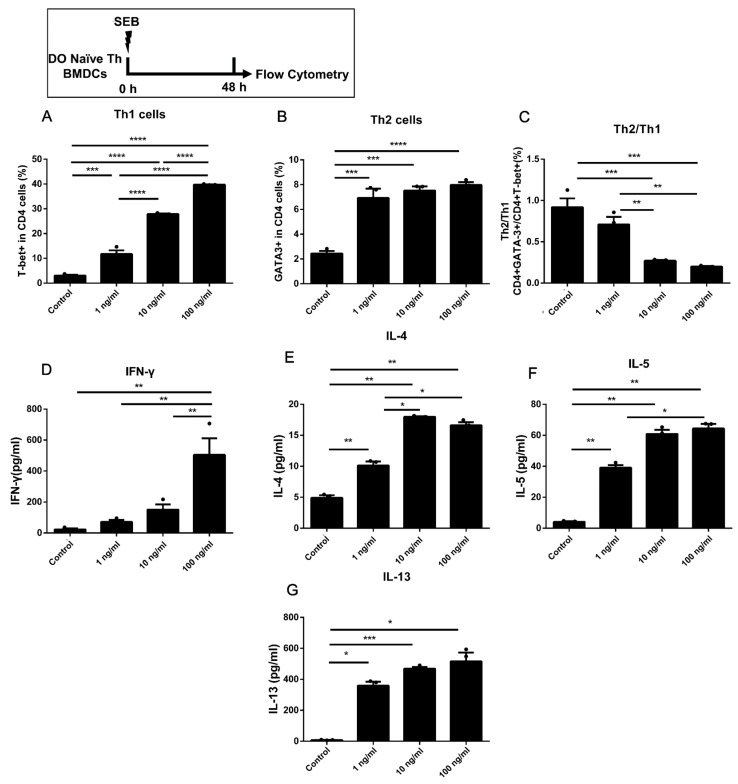
The Th1 and Th2 differentiation in DO11.10 naïve Th cells co-cultured with BMDCs under stimulation of SEB. (**A**,**B**) The percentages of T-bet+ and GATA-3+ in CD4 T cells. (**C**) The ratio of GATA-3/T-bet in CD4 T cells. The levels of IFN-γ (**D**), IL-4 (**E**), IL-5 (**F**), or IL-13 (**G**) are shown. Cells were stimulated with diluent (Control), 1 ng/mL SEB, 10 ng/mL SEB, and 100 ng/mL SEB (1 ng, 10 ng, or 100 ng SEB in each 1 mL medium) for 48 h. Data are represented with 3 technical replicates per group and presented as mean ± SEM. * *p* < 0.05, ** *p* < 0.01, *** *p* < 0.001, **** *p* < 0.0001.

**Figure 4:**
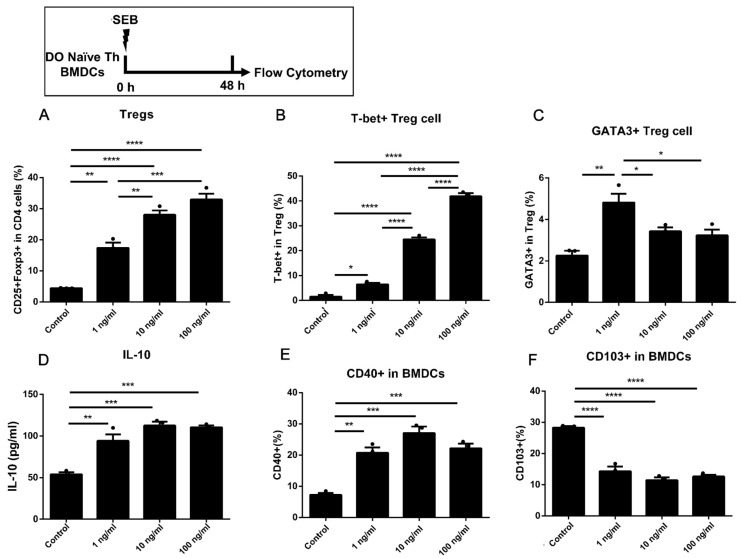
The Treg differentiation in DO11.10 naïve Th cells co-cultured with BMDCs under stimulation of SEB. (**A**) The percentage of Foxp3+ CD25+ in CD4 T cells. (**B**,**C**) The percentages of T-bet+ and GATA-3+ in CD25+Foxp3+ CD4 T cells. (**D**) The level of IL-10. (**E**,**F**) The expression of CD40 and CD103 in BMDCs. Cells were stimulated with diluent (Control), 1 ng/mL SEB, 10 ng/mL SEB, and 100 ng/mL SEB (1 ng, 10 ng, or 100 ng SEB in each 1 mL medium) for 48 h. Data are represented with 3 technical replicates per group and presented as mean ± SEM. * *p* < 0.05, ** *p* < 0.01, *** *p* < 0.001, **** *p* < 0.0001.

The naïve Th cells from DO11.10 mice were also involved in the co-culture model with BMDCs. The DO11.10 mice are homozygous ovalbumin (OVA)-TCR transgenic mice, which have a TCR that recognizes the 323–339 peptide fragment of OVA [28]. The percentages of T-bet, GATA-3, and Foxp3 in CD4 T cells increased under the stimulation of different doses of SEB compared to Control (Figure 3A,B and Figure 4A). Furthermore, the secretion of IL-4, IL-5, IL-13, and IL-10 was also increased under the stimulation of SEB compared to the Control (Figure 3E–G and Figure 4D). A dose-dependent increase in proportions of Th cells, Tregs, and Th1-like Tregs in CD4 T cells could be observed in SEB groups compared to the Control (Figure 3A and Figure 4A,B). The ratio of Th2/Th1 was decreased in the 10 and 100 ng/mL groups compared to the Control or 1 ng/mL group, respectively (Figure 3C). The proportion of Th2-like Tregs was higher in the Low group than in others (Figure 4C). Meanwhile, we also found a significant increase in CD40 and a decrease in CD103 in BMDCs co-cultured with DO11.10 Th cells (Figure 4E,F). The gating strategy of DCs is shown in Figure S1B.

### 2.4. The Activation of Naïve T Cells Could Be Induced by Co-Culture with BMDCs Pre-Stimulated with Higher Doses of SEB

**Figure 5:**
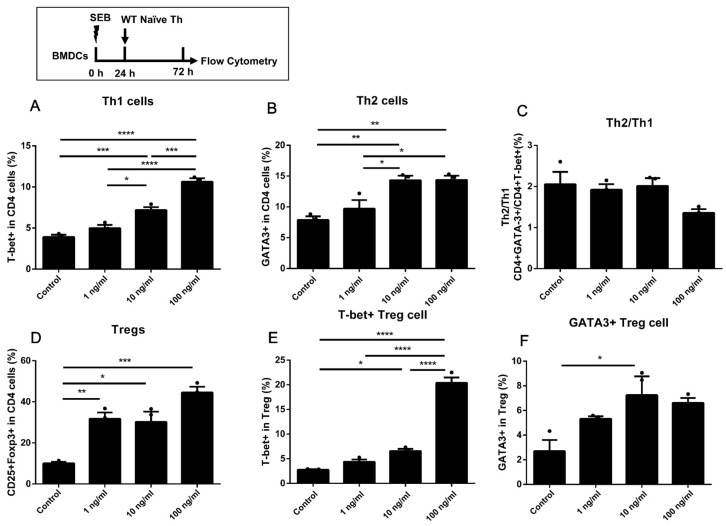
The differentiation of WT naïve Th cells co-cultured with BMDCs pre-stimulated with SEB. (**A**,**B**) The percentages of T-bet+ and GATA-3+ in CD4 T cells. (**C**) The ratio of GATA-3/T-bet in CD4 T cells. (**D**) The percentage of Foxp3+ CD25+ in CD4 T cells. (**E**,**F**) The percentages of T-bet+ and GATA-3+ in CD25+Foxp3+ CD4 T cells. The BMDCs were pre-stimulated with diluent (Control), 1 ng/mL SEB, 10 ng/mL SEB, and 100 ng/mL SEB (1 ng, 10 ng, or 100 ng SEB in each 1 mL medium) for 24 h. Then, the WT naïve Th cells were co-cultured with BMDCs for 48 h. Data are represented with 3 technical replicates per group and presented as mean ± SEM. * *p* < 0.05, ** *p* < 0.01, *** *p* < 0.001, **** *p* < 0.0001.

**Figure 6:**
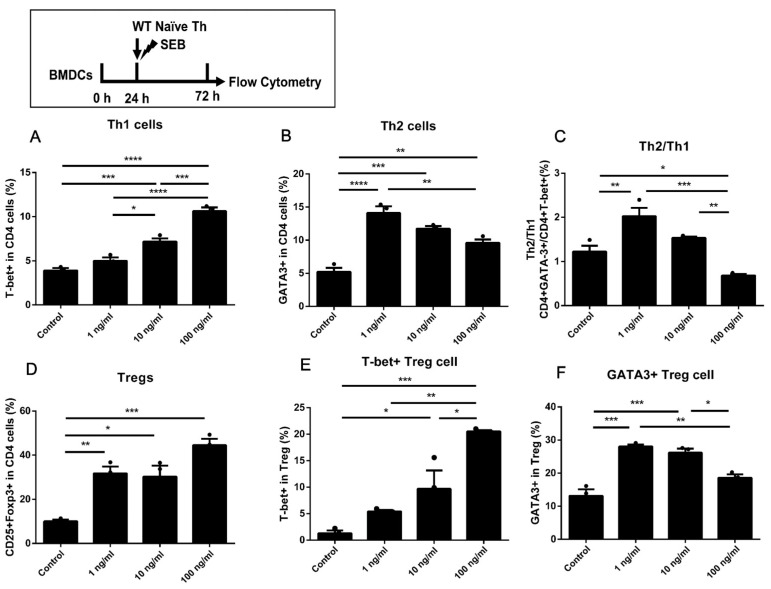
The differentiation of WT naïve Th cells co-cultured with BMDCs cultured alone. (**A**,**B**) The percentages of T-bet+ and GATA-3+ in CD4 T cells. (**C**) The ratio of GATA-3/T-bet in CD4 T cells. (**D**) The percentage of Foxp3+ CD25+ in CD4 T cells. (**E**,**F**) The percentages of T-bet+ and GATA-3+ in CD25+Foxp3+ CD4 T cells. Cells were stimulated with diluent (Control), 1 ng/mL SEB, 10 ng/mL SEB, and 100 ng/mL SEB (1 ng, 10 ng, or 100 ng SEB in each 1 mL medium) for 48 h. Data are represented with 3 technical replicates per group and presented as mean ± SEM. * *p* < 0.05, ** *p* < 0.01, *** *p* < 0.001, **** *p* < 0.0001.

The stimulation of SEB was introduced in the different phases of DC-T co-culture. In Figure 5, the BMDCs were pre-stimulated with SEB for 24 h and then co-cultured with naïve Th cells with SEB removed. A higher expression of T-bet and GATA-3 in CD4 T cells could only be found in BMDCs pre-stimulated with the higher doses of SEB (10 and 100 ng/mL) compared to the Control (Figure 5A,B). Additionally, there was no significant change in the Th2/Th1 ratio (Figure 5C) among groups. The 1 ng/mL of SEB could activate Treg differentiation (Figure 5D). T-bet expression in Tregs was only significantly increased in the 100 ng/mL group compared to the Control (Figure 6E). The stimulation of the 10 ng/mL dose of SEB led to higher expression of GATA-3 in Tregs than in the Control (Figure 5F).

### 2.5. More Th1 but Less Th2 Differentiation Was Found in the High Dose of SEB Group Compared to the Low Dose

In Figure 6, a time course similar to Figure 5 was adopted. The BMDCs were cultured alone for 24 h and then co-cultured with naïve Th cells under stimulation of SEB. The stimulation of SEB led to more proportions of Th1, Th2, and Treg cells than Control in all doses (Figure 6A,B,D). Additionally, a high dose of SEB (100 ng/mL) led to more expression of T-bet and less expression of GATA-3 in Th cells and Tregs compared to a low dose of SEB (1 ng/mL) (Figure 6B,F). Moreover, a significantly higher ratio of Th2/Th1 was only found in the 1 ng/mL group compared to the Control (Figure 6C). Additionally, a lower Th2/Th1 ratio was induced by a higher dose of SEB (100 ng/mL).

## 3. Discussion

*S. aureus* is one of the most common bacteria in individuals’ skin or gut flora and could generate enterotoxins leading to inflammation. It was reported that exposure to SEB is associated with several diseases, including infection, food allergy, asthma, and allergic rhinitis. As a superantigen, the plentiful activation of Th cells induced by SEB may contribute to these diseases [16,17]. Previous studies have found that the activation of Th2 cells in the high-dose SEB group was weaker than in the low-dose group [19,22]. It has been reported that in the food poisoning incidents caused by SEs, the content of SEB is 0.5–10μg in 100g of food and approximately equivalent to 5–100 ng SEB in 1 g of food [6]. To verify whether the dose of SEB might dominate the balance of Th differentiation and potentially contribute to inflammation or allergy, three doses of SEB (1, 10, and 100 ng/mL) were involved in the co-culture of BMDCs and naïve Th cells in this study. To characterize Th cell differentiation induced by SEB, the WT and DO11.10 naive Th cells were co-cultured with BMDCs. The differentiation of naive Th cells alone was also detected to determine whether DCs are required for SEB-induced Th differentiation. SEB stimulation was introduced at different stages of DC-T co-culture to determine whether the target cells of SEB were DCs or T cells.

Firstly, the proportions of Th1, Th2, and Treg cells and secretion of IFN-γ, IL-4, IL-5, or IL-13 in the DC-T co-culture model were all up-regulated by the stimulation of high-dose SEB (100 ng/mL). Under the stimulation of a low dose of SEB (1 ng/mL), there was no significant up-regulation of the Th1 proportion or IFN-γ secretion in WT Th cells (Figure 1A,D, Figure 2A, Figure 5A, and Figure 6A). The differentiation of Th2 cells was up-regulated by the 1 ng/mL SEB dose when the BMDCs were added in plates alone for 24 h (Figure 6B). Nevertheless, the ratio of Th2/Th1 in the 100 ng/mL group was partly decreased compared to the 1 ng/mL group. The promotion of Th1, Th2, and Treg cells in mice or cells induced by SEB with up-regulating cytokines was found, respectively, in previous studies [18,19,20,21,22,23]. As a superantigen, the extensive proliferation of T cells could be induced by the stimulation of SEB through the stable binding of CD28, β7, and TCR by SEB [24,29,30]. Previous literature has also indicated that different concentrations of SEB may lead to the undesired outcome that the Th cells in the high-dose SEB group are weaker than those in the low-dose group [22,29]. The proliferation ability of Th cells in the high-dose SEB group (500 ng/mouse) was more vulnerable than that in the low-dose group (50 ng/mouse) [24]. Additionally, 0–2 μg SEB led to distinct differentiation of Th1/Th2/Treg, which was regulated by the expression of Foxp3 [22]. It suggested that the stimulation of a high (100 ng/mL) concentration of SEB may lead to activation of Th1 and Treg but some suppression of Th2 immunity compared to a low dose (1 ng/mL) in our co-culture models of WT Th cells and BMDCs. The stimulation of naïve CD4+ T cells alone with SEB was designed to exclude the possible direct activation of Th cells by SEB. In our results, only 100 ng/mL SEB increased Th1 and Treg cells. This confirmed that Th differentiation dominated by different doses of SEB requires an APC presence. It may be due to the antagonization of over-activated Th1 and Treg differentiation [31]. The innovation in our work is robust evidence of Th differentiation induced by different doses of SEB in a DC-T co-culture cell model based on previous studies. Moreover, the skew towards Th2 differentiation induced by lower doses of SEB would be helpful in explaining the promotion of allergies induced by SEB [32,33].

The naïve Th cells isolated from DO11.10 mice carry the MHC class II-restricted rearranged T cell receptor transgene to OVA epitopes [34]. In DO11.10 Th cells, a more robust Th activation could be observed that up-regulated proportions of Th1, Th2, and Treg cells under stimulation with 1 ng/mL SEB (Figure 3A,B and Figure 4A) than that in WT Th cells (Figure 1). The T-bet expression in Th cells and Tregs increased, and these cells were dose-dependent when cultured with BMDCs (Figure 3). The secretions of IFN-γ and IL-10 could only be significantly increased under the stimulation of higher doses of SEB (10 and 100 ng/mL) compared to the Control (Figure 4). Such activation of Th immunity can also occur in allergen-specific Th cells. Th2 activation suggests that even low-dose SEB stimulation may promote the development of allergic diseases. The up-regulation of IFN-γ and IL-10 secretion in T cells induced by SEB has been confirmed by previous studies [23,35]. Our results further show that the levels of IL-10 and IFN-γ are dose-dependent on SEB in DO11.10 Th cells. Additionally, enhancement of Th1/Treg responses with more secretion of IFN-γ or IL-10 will be beneficial to restrict allergic diseases [36,37], leading to a decreased ratio of Th2/Th1 in the 100 ng/mL SEB group. Meanwhile, the stimulation of SEB led to increased expression of CD40 and a decrease in CD103 in BMDCs co-cultured with DO11.10 Th cells. The up-regulation of CD40 may be involved in activating naïve Th cells. Additionally, the expression of CD103 in DCs could be decreased by up-regulated IL-4 or IFN-γ and thus inhibits Tregs [38].

When co-cultured with BMDCs pre-stimulated with 10 and 100 ng/mL SEB, naïve Th cells could also be activated without SEB in the culture medium (Figure 5). However, the ratio of Th2/Th1 in higher dose groups (10 and 100 ng/mL) did not show significant changes compared to the Low or Control groups (Figure 5C). We speculate that the activation of Th cells in the absence of direct SEB stimulation might be due to the binding of SEB to MHC-Ⅱ molecules in BMDCs during the pre-stimulation. Another similar time course in which there was more Th2 differentiation was induced by 1 ng/mL SEB, while more Th1 and Treg differentiation was caused by 10 and 100 ng/mL SEB (Figure 6A,B,D). The proportion of Th2 and ratio of Th2/Th1 were significantly reduced in the 100 ng/mL SEB group compared to the 1 ng/mL (Figure 6B,C). This decline in GATA-3, similar to Figure 2, suggests that the BMDCs were functionally limited by preplacement in 48-well plates. Taken together with these results, we can speculate that the activation of naive Th cells is partially due to the co-culture with DCs and that the Th1/2 balance favoring Th1 caused by high doses of SEB requires direct stimulation of Th cells by SEB.

Among these results, the proportion of Tregs and the level of IL-10 could be significantly increased by 10 or 100 ng/mL SEB (Figure 1D, Figure 2D, Figure 3D, Figure 5D, and Figure 6D). However, this Treg up-regulation would be a negative feedback mechanism due to the significant up-regulation of Th1 or Th2 [27]. Further analyses also showed that the T-bet or GATA-3 expression trend in Tregs was similar to that in Th cells (Figure 2E,F, Figure 4B,C, and Figure 5E,F). These up-regulated Th-like Tregs, which expressed T-bet or GATA-3, could suppress the secretion of Th1 or Th2 cytokines [39]. Additionally, in vivo, exposure to SEB could lead to a partial decrease in the Treg proportion and immunosuppressive function [40,41].

## 4. Conclusions

In general, our results suggest that the balance of Th1/Th2 could be dominated by the doses of SEB stimulation. A higher SEB dose could induce more Th1 cells and a lower Th2/Th1 ratio. These findings expand and complement existing knowledge about SEB acting as a superantigen in Th cell activation. These findings are also helpful in understanding the role of *S. aureus* colonization and SEB contamination in immune-mediated diseases.

## 5. Materials and Methods

### 5.1. Generation of BMDCs and Isolation of Spleen Naïve CD4 T Cells

DCs were generated from bone marrow cells of 5- to 7-week-old female BALB/C mice [42]. Briefly, BM cells were recovered from the femurs and tibias and cultured in a complete culture medium (RPMI 1640 supplemented with 10% fetal bovine serum, all purchased from Cellmax, Sunnyvale, CA, USA) containing 20 ng/mL GM-CSF and 10 ng/mL IL-4 (Peprotech, Cranbury, NJ, USA) for 7 days at 37 °C. Half of the medium was discarded and replaced with an equal-volume fresh medium on the third and sixth days.

Naïve CD4 T cells were isolated from the spleen of WT BALB/C mice and DO11.10 mice (Strain #:003303, purchased from The Jackson Lab, Bar Harbor, ME, USA) using a Naïve CD4 T Cell Isolation Kit (Miltenyi Biotec, Cologne, Germany). The spleens were ground and incubated in a Red Blood Cell Lysis Buffer (Solarbio) for 5 min to release lymphocytes. Magnetic bead sorting operation of naïve CD4 T cells was performed according to the instructions of the isolation kit. The splenic lymphocytes were incubated with a magnetic bead cocktail. The uncaptured cells passing through the column were the target Th cells for the subsequent co-culture.

All animal experiments and handling procedures were approved by the Animal Care and Use Committees of Nanchang University and were performed in accordance with institutional guidelines (Animal ethics code: 2021-0308-035).

### 5.2. Cell Stimulation

The naïve Th cells (5 × 10^5^ cells in the 1 mL medium) isolated from WT mice were stimulated with diluent (Control), 1 ng/mL SEB (purchased from Toxin Technology, Inc., Florida, USA. Catalog # BT202), 10 ng/mL SEB, and 100 ng/mL SEB (1 ng, 10 ng, or 100 ng SEB in each 1 mL medium) for 48 h in a 48-well plate, with or without the presence of BMDCs (2.5 × 10^5^ cells in the 1 mL medium). The naïve Th cells isolated from DO11.10 mice were also stimulated with diluent (Control) or different doses of SEB with BMDCs. The culture volume of each sample was 1 mL. In other groups, BMDCs were first stimulated with different doses of SEB and then co-cultured with Th cell spines.

### 5.3. Flow Cytometry and Antibodies

Cells were collected and resuspended with a staining buffer (PBS with 2% FBS) for flow cytometry. Cells were firstly blocked with anti-CD16/32 (Fcblock, ThermoFisher, Waltham, MA, USA) and stained with 0.1% Fixable Viability Stain 510 (BD Biosciences, Franklin Lakes, NJ, USA) for 30 min at 4 °C to block Fc receptors and distinguish dead cells. Anti-mouse antibodies, including BV605-CD11c (N418), FITC-I-A/I-E (M5/114.15.2), PE/Cyanine7-CD103 (2E7), and PerCP/Cyanine5.5-CD40 (3/23) (all purchased from Biolegend, San Diego, CA, USA), were used to stain DCs for 30 min at 4 °C. For Th cells, BB515-CD25 (PC61, BD) and BV605-CD4 (RM4-5, Biolegend, San Diego, CA, USA) were used. A BD Pharmingen™ Transcription Factor Buffer (BD Biosciences, Franklin Lakes, NJ, USA) was used to detect the transcription factor in Th cells. Th cells were incubated with a fixation/permeabilization buffer for 45 min at 25 °C and washed with a permeabilization/wash buffer twice. Additionally, Th cells were stained with AF647-T-bet (04-46, BD), BV421-GATA-3 (L50-823, BD Biosciences, Franklin Lakes, NJ, USA), and PE-Cyanine7-Foxp3 (PC61) (ThermoFisher, Waltham, MA, USA) for 45 min at 4 °C. The working concentration of each antibody is referred to in the respective manual.

The data were obtained with a CytoFLEX Flow Cytometer (Beckman Coulter, Brea, CA, USA) and rendered using FlowJo software (FlowJo V10.6.2, Becton Dickinson & Company, Franklin Lakes, NJ, USA). The placement of gates was determined by Fluorescence Minus One method.

### 5.4. ELISAs

The supernatant was collected after stimulation to detect the secretions of IL-4, IL-5, IL-10, IL-13, and IFN-γ through ELISA kits (Invitrogen, Thermo Fisher Scientific, Waltham, MA, USA). Undiluted culture supernatants were assayed after centrifugation and stored at −80 °C for a week. The procedure was performed according to the manufacturer’s instructions. The 96-well plates were coated with 100 μL/well of capture antibodies of cytokines overnight at 4 °C. After blocking with 200 μL/well assay buffer for 1 h, 100 μL/well of the supernatant or 2-fold serial dilution standards were added and incubated in room temperature for 2 h. The plate was then incubated for 1 h with 100 μL/well of a diluted detection antibody, and then 100 mL of diluted Streptavidin-HRP was added in each well. The plates were read at 450 nm after incubating with 100 μL/well of a TMB solution for 15 min and stopped with a 1 M H_2_SO_4_ solution. According to the kit, the standard curve is linear. The standard curve range of IL-4, IL-5, and IL-13 is 4–500 pg/mL, 16–2000 pg/mL for IFN-γ, and 32–4000 pg/mL for IL-10.

### 5.5. Statistical Analysis

All data were analyzed using GraphPad Prism 6 (GraphPad Software, Inc., Dotmatics, MA, USA). Statistical significance was determined with a one-way ANOVA with Tukey’s multiple comparison tests (* *p* < 0.05, ** *p* < 0.01, *** *p* < 0.001, **** *p* < 0.0001).

## Data Availability

The data presented in this study are available on request from the corresponding author.

## Supplementary Materials

The following supporting information can be downloaded at: https://www.mdpi.com/article/10.3390/toxins15060363/s1, Figure S1: The gating strategy of Th and DC cells.; Figure S2: The model diagrams of cell stimulation.
