# Supplementary material for: A Higher Dose of Staphylococcus aureus Enterotoxin B Led to More Th1 and Lower Th2/Th1 Ratio in Th Cells
Source: Toxins (Basel). 2023 May 28;15(6):363. doi: 10.3390/toxins15060363 (PMC10302221; doi:10.3390/toxins15060363)
Supplement: Supplementary file 1 [file toxins-15-00363-s001.zip › toxins-2322100-supplementary.pdf]

Supplementary Materials:

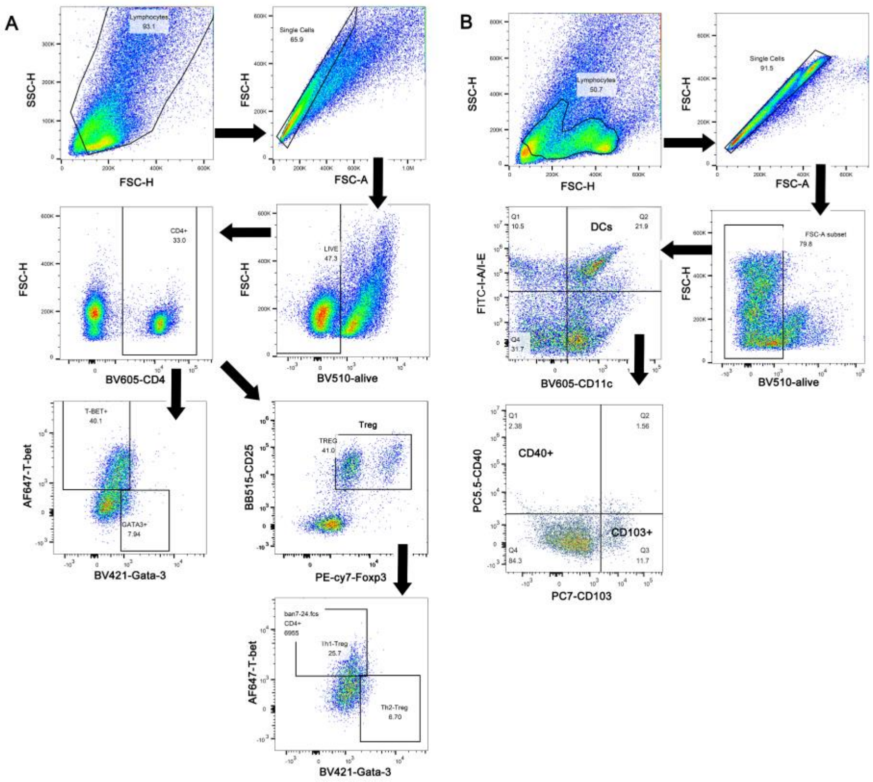

Figure S1. (A) The gating strategy of Th cells. (B) The gating strategy of DCs.

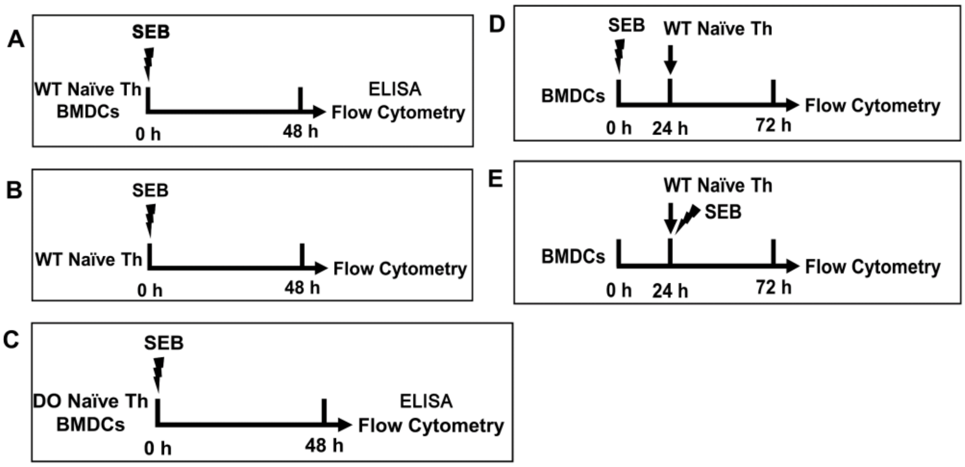

Figure S2. The model diagrams of cell stimulation. (A) WT naïve Th cells were co-cultured with BMDCs under stimulation of different doses of SEB. (B) WT naïve Th cells were stimulated with different doses of SEB. (C) WT naïve Th cells were co-cultured with BMDCs under stimulation of different doses of SEB. (D) BMDCs were pre-stimulated with different doses of SEB and then co-cultured with WT naïve Th cells. (E) BMDCs were pre-cultured in the plate and then co-cultured with WT naïve Th cells under stimulation of different doses of SEB.
